# Dataset on the relationships between flipped classroom approach, students’ learning satisfaction and online learning anxiety in the context of Saudi Arabian higher education institutions.

**DOI:** 10.1016/j.dib.2022.108588

**Published:** 2022-09-15

**Authors:** Turki Mesfer Alqahtani, Farrah Dina Yusop, Siti Hajar Halili

**Affiliations:** a Department of Curriculum and Instructional Technology, Faculty of Education, Universiti Malaya, Kuala Lumpur, Malaysia; b Department of Instructional Technology, Faculty of Education, Jazan University, Jazan, Saudi Arabia

**Keywords:** Flipped classroom, Online learning anxiety, Higher education, learning satisfaction, Saudi Arabia, FC, Flipped Classroom, OLA, Online Learning Anxiety

## Abstract

This paper presents data from 417 undergraduate students on the relationship between the flipped classroom (FC) approach and students' learning satisfaction (SAT), as well as the impact of online learning anxiety (OLA) in the context of Saudi Arabian higher education. A 5-Likert scale questionnaire with 74 items on students' demographic information, flipped classroom approach, learning satisfaction, and online learning anxiety was used to collect the data. The dataset will be a valuable resource for policymakers, stakeholders, and academics seeking to better understand the most effective ways to implement the flipped classroom approach.

**Specifications Table**

**Table:** 

Subject	Social Sciences
Specific subject area	Educational Technology
Type of data	Table
How the data were acquired	The data were acquired through an online survey. The questionnaire is available at https://data.mendeley.com/datasets/5jdd74ggz8/6
Data format	Raw, Analyzed
Description of data collection	The questionnaire was distributed to 417 undergraduate students at four Saudi universities using systematic sampling procedures. The universities that were chosen have the most enrolled students, represent the north, south, west, and east of Saudi Arabia, and have agreed to take part in the study. Their participation was completely voluntary, anonymous and did not include any information that could have revealed their identities to protect their privacy.
Data source location	Institutions: Jazan University (JU); University of Tabuk (UT); University of Jeddah (UJ); Imam Abdulrahman Bin Faisal University (IAU). Regions: North, South, West and East regions. Country: Saudi Arabia
Data accessibility	Repository name: Mendeley Data Data identification number: 10.17632/5jdd74ggz8.2 Direct URL to data: https://data.mendeley.com/datasets/5jdd74ggz8/6

## Value of the Data

• The dataset provides a better understanding and insights into the relationships between the flipped classroom approach, students' learning satisfaction, and online learning anxiety in the context of Saudi higher education.
• The availability of this dataset as an open access dataset will provide policymakers, stakeholders, and academics with a better understanding of the role of online learning anxiety, allowing them to make more informed decisions about implementing the flipped classroom approach.
• The dataset can be used by educational researchers to compare it to data from similar studies conducted in other regions or universities.
• This dataset can also be used to conduct a new analysis comparing student satisfaction in the flipped classroom and traditional teaching methods.

## 1 Data Description

This dataset contains information gathered from an online questionnaire distributed to 417 undergraduate students at four Saudi universities spread across the Kingdom of Saudi Arabia. The dataset can be found at Mendeley Data [1].

The questionnaire consisted of 74 items on a five-point Likert scale (1 = Strongly Disagree, 2 = Disagree, 3 = Neither, 4 = Agree, 5 = Strongly Agree) and was divided into four sections. Section 1 contains demographic information about participants (5 items), Section 2 contains information about the Flipped Classroom (FC) approach (31 items), Section 3 contains information about students' learning satisfaction (31 items), and Section 4 contains information about students' online learning anxiety (12 items). The variables, codes, definitions, and references of the adapted scales for Sections 2 to 4 of the questionnaire are shown in Table 1.

**Table 1:** Variables, codes, definitions, and references of the adapted scales.

Variables	Code	Definition	References of the adapted scales
1. Flipped Classroom	FC	A teaching approach that provides lecture content through online platforms outside of class and class time is used for interactive learning and communication with peers.	[2]
2. Satisfaction	SAT	A positive impression of learning activities and experiences in the learning environment.	[3]
3. Online Learning Anxiety	OLA	The fear and uncomfortable feelings that students experience when learning online.	[4]

Table 2 presents the demographic information of the participants (Section 1) based on five items: age, gender, institute, specialisation (i.e. academic major), and academic year. Out of the 417 participants, a total of 202 (48.4%) of the respondents were male, while the rest of the respondents 215 (51.6%) were female. In terms of the age of the respondents, 223 (53.5%) of them are between 21 to 23 years old. 108 (25.9%) of the total respondents were aged between 18 to 20 years, while 63 (15.1%) were aged between 24 to 26 years. Respondents over 26 years old made up 5.5% of the total response. Hence, the majority of respondents were between 21 and 23 years old, and the minority of them were over 26 years old.

**Table 2:** Demographic profile of the respondents.

No	Demographic Item	Code	Categories	Frequency (n=417)	Percentage %
1	Gender	1	Male	202	48.4
		2	Female	215	51.6

2	Age	1	18 to 20 years old	108	25.9
		2	21 to 23 years old	223	53.5
		3	24 to 26 years old	63	15.1
		4	More than 26 years old	23	5.5

3	Institute	1	Jazan University (JU)	168	40.3
		2	University of Tabuk (UT)	101	24.2
		3	University of Jeddah (UJ)	37	8.9
		4	Imam Abdulrahman Bin Faisal University (IAU)	111	26.6

4	Major or specialisation	1	Humanity and Social Sciences	51	12.2
		2	Medicine and Medical Services	47	11.3
		3	Engineering and Engineering Industries	55	13.2
		4	Education Sciences	64	15.3
		5	Sharia Sciences and Islamic Studies	39	9.4
		6	Computer Science and Information Technology	47	11.3
		7	Science of Management, Business and Law	64	15.3
		8	Sciences	50	12.0

5	Academic Year	1	First Year	60	14.4
		2	Second Year	60	14.4
		3	Third Year	92	22.1
		4	Fourth Year	155	37.2
		5	Fifth Year	50	12.0

Further, Table 2 displays that the highest number of participants were from Jazan University (JU), followed by students from Imam Abdulrahman bin Faisal University (IAU), University of Tabuk (UT) and University of Jeddah (UJ).

The majority of the students are from the fields of Education Sciences (15.3%), and Science of Management, Business and Law (15.3%), followed by students from the Engineering and Engineering Industries (13.2%), Humanity and Social Sciences (12.2%), and Sciences respectively (12.0%). The lowest participation was students from the field of Sharia Sciences and Islamic Studies (9.4%).

In terms of the academic year, the majority of respondents were in their fourth year, followed by those in their third. Sixty students are in their first and second years, with the majority of respondents in their fifth year. All students completed 74 items from the three main constructs: the FC, the SAT, and the OLA.

Sections 2 to 4 of the questionnaire include data from all 74 items of each construct (FC, SAT, and OLA), which were further classified based on their dimensions, as shown in Table 3. Values of skewness and kurtosis of all dimensions are presented in Table 4. The complete questionnaire, which is available from the Mendeley dataset, contains more information on the items.

**Table 3:** The constructs, codes, dimensions, and number of items of the questionnaire.

Construct	Codes	Dimensions	Number of items
Flipped Classroom	FKC	Knowledge Construction	5
(FC)	FIL	In-Depth Learning	4
	FAU	Authenticity	4
	FMP	Perspective	3
	FPK	Prior Knowledge	4
	FTS	Lecturer-Student Interaction	5
	FSI	Social Interaction	6
	FCD	Cooperative Dialogue	

Satisfaction	SIT	Interaction	9
(SAT)	SIS	Instruction	12
	SIO	Instructor	5
	STG	Technology	5

Online Learning Anxiety	OCA	Computer Anxiety	5
(OLA)	OCX	Communication Anxiety	3
	OOX	Online Learning Anxiety	4

**Table 4:** Values of Skewness and Kurtosis of all dimensions.

Variables	Dimensions	Skewness	Kurtosis
Flipped Classroom	FKC	-.381	-.652
(FC)	FIL	-.641	-.713
	FAU	-.639	-.679
	FMP	-.457	-.725
	FPK	-.769	-.567
	FTS	-.608	-.915
	FSI	-.503	-.759
	FCD	-.596	-.724

Satisfaction	SIT	-.452	-.540
(SAT)	SIS	-.381	-.820
	SIO	-.391	-.783
	STG	-.390	-.845

Online Learning Anxiety	OCA	.286	-.853
(OLA)	OCX	.298	-.755
	OOX	-.155	-1.141

## 2 Experimental Design, Materials and Methods

The purpose of this research was to investigate the relationship between the flipped classroom (FC) approach and students' learning satisfaction, as well as the role of online learning anxiety in this relationship, in the context of Saudi Arabia's higher education institutions. This study included undergraduate students from four universities in four different regions of the Kingdom of Saudi Arabia. Eight hundred questionnaires were distributed to students online, but only 417 completed questionnaires were returned. The information gathered was coded and analysed using Partial Least Squares Structural Equation Modelling (PLS-SEM).

Furthermore, data reliability was assessed using Cronbach's alpha. Table 5 shows that the Cronbach alpha (α) value of the dimensions in the three reflective variables ranges from .0829 to 0.941 (>0.7), while the Composite Reliability value ranges from 0.898 to 0.951 (>0.7), which are both acceptable and eligible for further analysis [5]. Furthermore, the AVE value of the dimensions in the three reflective variables range is greater than the acceptable value of 0.5 [5].

**Table 5:** Composite reliability and Average Variance Extracted (AVE) of variables.

Variables	Dimensions	Cronbach alpha (α)	Composite Reliability	Average Variance Extracted (AVE)
**FC**	**FKC**	0.910	0.933	0.736
	**FIL**	0.898	0.929	0.767
	**FAU**	0.885	0.921	0.743
	**FMP**	0.852	0.911	0.774
	**FPK**	0.883	0.920	0.741
	**FTS**	0.904	0.929	0.724
	**FSI**	0.882	0.927	0.809
	**FCD**	0.890	0.931	0.819

**SAT**	**SIT**	0.906	0.928	0.682
	**SIS**	0.941	0.951	0.710
	**SIO**	0.917	0.942	0.801
	**STG**	0.891	0.919	0.696

**OLA**	**OCA**	0.907	0.931	0.730
	**OCX**	0.829	0.898	0.747
	**OOX**	0.882	0.919	0.740

Table 6 revealed that some items were deleted because their outer loading values were less than 0.708. Outer loadings are the results of single regressions on each indicator variable's corresponding construct [5]. It is also known as the indicator reliability and indicates the items' commonality. It also refers to the extent to which the construct defines the variance in the items. Because of this deletion, the CR and AVE values can be kept within acceptable limits [5]. The following items were removed from the scale: SIT34, SIT36, SIT38, SIS43, SIS45, SIS48, SIS51, and SIO54.

**Table 6:** Results of the outer loadings, for all variables.

Variable	Dimensions	Items	Loading (>0.7)	Variable	Dimensions	Items	Loading (>0.7)
FC	FKC	FKC1	**0.870**			SIT39	**0.837**
		FKC2	**0.887**			SIT40	**0.781**
		FKC3	**0.915**		SIS	SIS41	**0.813**
		FKC4	**0.815**			SIS42	**0.816**
		FKC5	**0.800**			SIS43	**0.625**
	FIL	FIL6	**0.881**			SIS44	**0.844**
		FIL7	**0.901**			SIS45	**0.501**
		FIL8	**0.912**			SIS46	**0.845**
		FIL9	**0.805**			SIS47	**0.845**
	FAU	FAU10	**0.864**			SIS48	**0.527**
		FAU11	**0.883**			SIS49	**0.820**
		FAU12	**0.873**			SIS50	**0.866**
		FAU13	**0.827**			SIS51	**0.610**
	FMP	FMP14	**0.908**			SIS52	**0.806**
		FMP15	**0.920**		SIO	SIO53	**0.853**
		FMP16	**0.806**			SIO54	**0.608**
	FPK	FPK17	**0.801**			SIO55	**0.910**
		FPK18	**0.816**			SIO56	**0.891**
		FPK19	**0.917**			SIO57	**0.886**
		FPK20	**0.904**		STG	STG58	**0.870**
	FTS	FTS21	**0.866**			STG59	**0.901**
		FTS22	**0.887**			STG60	**0.833**
		FTS23	**0.814**			STG61	**0.785**
		FTS24	**0.876**			STG62	**0.776**
		FTS25	**0.808**	OLA	OCA	OCA63	**0.811**
	FSI	FSI26	**0.878**			OCA64	**0.866**
		FSI27	**0.904**			OCA65	**0.887**
		FSI28	**0.916**			OCA66	**0.873**
	FCD	FCD29	**0.890**			OCA67	**0.831**
		FCD30	**0.921**		OCX	OCX68	**0.897**
		FCD31	**0.905**			OCX69	**0.809**

SAT	SIT	SIT32	**0.810**			OCX70	**0.884**
		SIT33	**0.857**		OOX	OOX71	**0.790**
		SIT34	**0.687**			OOX72	**0.899**
		SIT35	**0.824**			OOX73	**0.900**
		SIT36	**0.571**			OOX74	**0.846**
		SIT37	**0.760**				
		SIT38	**0.469**				

These items were removed from the scale because of low loadings, where the value of each removed item was 0.687, 0.571, 0.469, 0.625, 0.501, 0.527, 0.610, and 0.608, respectively. In addition, Table 7 presents Fornell-Larcker's criterion which is a second technique to measure the discriminant validity of the scale. This technique compares the square root of the AVE values with its relationship of the latent variables (other dimensions) [5].

**Table 7:** The square root of the average variance extracted.

		**FKC**	**FIL**	**FAU**	**FMP**	**FPK**	**FTS**	**FSI**	**FCD**	**SIT**	**SIS**	**SIO**	**STG**	**OCA**	**OCX**	**OOX**
**FC**	**FKC**	**0.858**														
	**FIL**	0.748	**0.876**													
	**FAU**	0.645	0.761	**0.862**												
	**FMP**	0.676	0.703	0.770	**0.880**											
	**FPK**	0.650	0.697	0.672	0.777	**0.861**										
	**FTS**	0.611	0.697	0.668	0.747	0.807	**0.851**									
	**FSI**	0.579	0.648	0.600	0.654	0.669	0.755	**0.899**								
	**FCD**	0.619	0.673	0.667	0.686	0.669	0.732	0.797	**0.905**							

**SAT**	**SIT**	0.650	0.677	0.650	0.681	0.731	0.726	0.664	0.668	**0.744**						
	**SIS**	0.685	0.700	0.694	0.734	0.751	0.751	0.710	0.739	0.775	**0.754**					
	**SIO**	0.661	0.667	0.670	0.712	0.704	0.739	0.660	0.708	0.694	0.787	**0.837**				
	**STG**	0.599	0.603	0.591	0.625	0.632	0.678	0.623	0.655	0.669	0.717	0.749	**0.834**			

**OLA**	**OCA**	0.139	0.177	0.122	0.150	0.183	0.215	0.183	0.137	0.162	0.127	0.095	0.162	**0.854**		
	**OCX**	0.042	0.086	0.056	0.056	0.093	0.090	0.072	0.050	0.019	0.043	0.016	0.015	0.566	**0.864**	
	**OOX**	0.172	0.141	0.123	0.144	0.150	0.132	0.140	0.162	0.200	0.230	0.246	0.269	0.433	0.521	**0.860.**

Table 7 illustrates that the square root of the value of AVE of each dimension is not bigger than its highest relationship with other dimensions. These items were removed from the scale due to low loadings, with values of 0.687, 0.571, 0.469, 0.625, 0.501, 0.527, 0.610, and 0.608 respectively. Table 7 also includes Fornell-Larcker's criterion, which is a second technique for determining the discriminant validity of the scale. This method compares the square root of the AVE values to their relationship with the latent variables (other dimensions) [5]. As shown in Table 7, the square root of each dimension's AVE value is not greater than its highest relationship with other dimensions.

## Ethics Statement

Prior to data collection, participants had been informed that their participation in this non-experimental research was entirely voluntary and that they could leave at any time and for any reason, with no penalty or loss of benefits to which they were entitled, if any. They were also informed that there were no known risks associated with participating in the research. Furthermore, the content of their survey will be kept private, and the data collected will not contain any information that could be used to identify the students. All participants have voluntarily consented to participate.

## CRediT authorship contribution statement

**Turki Mesfer Alqahtani:** Data curation, Writing – original draft. **Farrah Dina Yusop:** Writing – review & editing, Conceptualization, Methodology, Supervision, Funding acquisition. **Siti Hajar Halili:** Supervision.

## Declaration of Competing Interest

The authors declare that they have no known competing financial interests or personal relationships that could have appeared to influence the work reported in this paper.

## Data Availability

The Moderating Role of OLA on The Relationship Between FC and SAT (Original data) (Mendeley Data).

## Appendix Supplementary materials

Image, application 1

Image, application 2
