# Supplementary material for: Dataset on the relationships between flipped classroom approach, students’ learning satisfaction and online learning anxiety in the context of Saudi Arabian higher education institutions
Source: Data Brief. 2022 Sep 15;45:108588. doi: 10.1016/j.dib.2022.108588 (PMC9519427; doi:10.1016/j.dib.2022.108588)
Supplement: Supplementary file 1 [file mmc1.docx]

**Informed Consent**

Dear respondents,

This research is being conducted to examine the relationship between learning experience and motivation, satisfaction in the flipped classroom environment. If you give permission to participate, you will be answering a survey that could take 10-15 minutes.

**RISKS**

There are no foreseeable risks for participating in this research.

**BENEFITS**

There are no direct benefits to you as a participant. However, this study could provide positive insights about the flipped classroom in higher education institutes.

**CONFIDENTIALITY**

The content of your survey will remain confidential. Your name or any individual identifiable will not be included on collected data. Only the researcher will have access to the data.

**PARTICIPATION**

Your participation is voluntary, and you may withdraw from the study at any time and for any reason. If you decide not to withdraw from the study, there is no penalty or loss of benefits to which you are otherwise entitled. There are no costs to you or any other party.

**CONTACT**

This research is being conducted by Turki Alqahtani, a PhD student at University of Malaya. He may be reached at [turki.mf.h@gmail.com](mailto:turki.mf.h@gmail.com) . This research is being supervised by:

Dr. Farrah Dina Yusop

Department of Curriculum and Instructional Technology,

Faculty of Education, University of Malaya

[farah@um.edu.my](mailto:farah@um.edu.my)

Dr. Siti Hajar Halili

Department of Curriculum and Instructional Technology,

Faculty of Education, University of Malaya

[siti_hajar@um.edu.my](mailto:siti_hajar@um.edu.my)

Thank you for your time and co-operation
