# Supplementary material for: Dataset on the relationships between flipped classroom approach, students’ learning satisfaction and online learning anxiety in the context of Saudi Arabian higher education institutions
Source: Data Brief. 2022 Sep 15;45:108588. doi: 10.1016/j.dib.2022.108588 (PMC9519427; doi:10.1016/j.dib.2022.108588)
Supplement: Supplementary file 2 [file mmc2.docx]

## **Questionnaire**

**Part A: General Demographic Information of the Participants**

**Please chose an appropriate response for you:**

| **Institute** | |
| --- | --- |
| 🞎 Jazan University (JU) | 🞎 University of Jeddah (UJ) |
| 🞎 Imam Abdulrahman Bin Faisal University (IAU) | 🞎 University of Tabuk (UT) |
| **Year of Study** | |
| 🞎 First Year | |
| 🞎 Second Year | |
| 🞎 Third Year | |
| 🞎 Forth Year | |
| 🞎 Fifth Year | |
| **Faculty :** | |
| **Major:** | |
| **Gender:** 🞎 Male 🞎 Female | |
| **Age:** | |
| 🞎 18-20 | |
| 🞎 21-23 | |
| 🞎24-26 | |
| 🞎 over 26 | |

Instruction: Please rate the following statements based on the scale below.

| 1 | 2 | 3 | 4 | 5 |
| --- | --- | --- | --- | --- |
| Strongly Disagree | Disagree | Neutral | Agree | Strongly Agree |
| SD | D | N | A | SA |

**Part B: Learning Experience in The Flipped Classroom**

| No | **In This course ….** | SD | D | N | A | SA |
| --- | --- | --- | --- | --- | --- | --- |
| 1 | In this course, I was given opportunities to investigate real problems | 1 | 2 | 3 | 4 | 5 |
| 2 | In this course, I was given opportunities to raise questions about complex problems | 1 | 2 | 3 | 4 | 5 |
| 3 | In this course, I was given opportunities to search for possible explanations for real problems | 1 | 2 | 3 | 4 | 5 |
| 4 | In this course, I was asked to analyse data regarding a significant problem I have raised during this course | 1 | 2 | 3 | 4 | 5 |
| 5 | In this course, I was asked to draw conclusions from a research work, in which I have participated | 1 | 2 | 3 | 4 | 5 |
| 6 | In this course, I have learned skills with which I can deeply explore a subject which is of interest to me | 1 | 2 | 3 | 4 | 5 |
| 7 | In this course, I could examine in depth a major issue | 1 | 2 | 3 | 4 | 5 |
| 8 | In this course, I have focused on a central subject which I was required to deeply understand | 1 | 2 | 3 | 4 | 5 |
| 9 | In this course, I have learned how to investigate intensely a certain subject | 1 | 2 | 3 | 4 | 5 |
| 10 | This course addressed interesting situations in reality | 1 | 2 | 3 | 4 | 5 |
| 11 | This course focused on giving relevant meaning to the learned concepts | 1 | 2 | 3 | 4 | 5 |
| 12 | This course addressed real life and interesting events | 1 | 2 | 3 | 4 | 5 |
| 13 | This course was rich with real-life examples that interest me | 1 | 2 | 3 | 4 | 5 |
| 14 | In this course, ideas were presented from several point of view | 1 | 2 | 3 | 4 | 5 |
| 15 | In this course, I have learned about complex real issues | 1 | 2 | 3 | 4 | 5 |
| 16 | In this course, I had to question and criticise accepted ideas | 1 | 2 | 3 | 4 | 5 |
| 17 | This course dealt with subjects I have learned in other courses | 1 | 2 | 3 | 4 | 5 |
| 18 | In this course, the subjects learned were related to prior knowledge I have gained | 1 | 2 | 3 | 4 | 5 |
| 19 | In this course, the things that I have learned have helped me understand issues which I have learned in other courses | 1 | 2 | 3 | 4 | 5 |
| 20 | In this course, the subjects were related to diverse contents of knowledge | 1 | 2 | 3 | 4 | 5 |
| 21 | In this course, the lecturer allowed me to think about my learning and how to improve it | 1 | 2 | 3 | 4 | 5 |
| 22 | In this course, the lecturer considered my learning pace | 1 | 2 | 3 | 4 | 5 |
| 23 | In this course, I could set myself some learning goals | 1 | 2 | 3 | 4 | 5 |
| 24 | In this course, the lecturer encouraged me to think about my learning and ways to improve it | 1 | 2 | 3 | 4 | 5 |
| 25 | In this course, the lecturer made me think about the advantages and disadvantages of my learning | 1 | 2 | 3 | 4 | 5 |
| 26 | This course included a variety of learning activities with other students | 1 | 2 | 3 | 4 | 5 |
| 27 | In this course, I was given opportunities to learn with other students | 1 | 2 | 3 | 4 | 5 |
| 28 | In this course, I could collaborate with other students | 1 | 2 | 3 | 4 | 5 |
| 29 | In this course, Arguments and discussions were held | 1 | 2 | 3 | 4 | 5 |
| 30 | In this course, it was possible to express original ideas | 1 | 2 | 3 | 4 | 5 |
| 31 | In this course, I could express my opinion, even when it was different from other students | 1 | 2 | 3 | 4 | 5 |

**Part C: Student Satisfaction**

| No | **In this course…** | SD | D | N | A | SA |
| --- | --- | --- | --- | --- | --- | --- |
| 32 | A flipped classroom session keeps me always alert and focused. | 1 | 2 | 3 | 4 | 5 |
| 33 | In this course, interaction is adequately maintained with the lecturer | 1 | 2 | 3 | 4 | 5 |
| 34 | In this course, having students from the opposite gender listening to what I say might restrict my participation. | 1 | 2 | 3 | 4 | 5 |
| 35 | A flipped classroom course makes it more important for students to visit the lecturer during office-hours. | 1 | 2 | 3 | 4 | 5 |
| 36 | In this course, I cannot interrupt the lecturer to ask a question | 1 | 2 | 3 | 4 | 5 |
| 37 | In this course, I am satisfied with the quality of interaction between all involved parties. | 1 | 2 | 3 | 4 | 5 |
| 38 | In this course, I am dissatisfied with the process of collaboration activities during the course. | 1 | 2 | 3 | 4 | 5 |
| 39 | In this course, I am satisfied with the way I interact with other students. | 1 | 2 | 3 | 4 | 5 |
| 40 | In this course, I am satisfied with my participation in the class. | 1 | 2 | 3 | 4 | 5 |
| 41 | In this course, the use of the flipped classroom approach encourages me to learn independently. | 1 | 2 | 3 | 4 | 5 |
| 42 | In this course, my understanding is improved compared to similar courses I studied before. | 1 | 2 | 3 | 4 | 5 |
| 43 | In this course, my performance in exams is improved compared to similar courses I studied before. | 1 | 2 | 3 | 4 | 5 |
| 44 | In this course, I am satisfied with the level of effort this course required. | 1 | 2 | 3 | 4 | 5 |
| 45 | In this course, I am dissatisfied with my performance in this course. | 1 | 2 | 3 | 4 | 5 |
| 46 | In this course, I believe I will be satisfied with my final grade in the course. | 1 | 2 | 3 | 4 | 5 |
| 47 | In this course, I am satisfied with how I am able to apply what I have learned in this course. | 1 | 2 | 3 | 4 | 5 |
| 48 | If I had known this was going to be a flipped classroom class, I would not have taken it. | 1 | 2 | 3 | 4 | 5 |
| 49 | I am willing to take another course using the flipped classroom delivery mode. | 1 | 2 | 3 | 4 | 5 |
| 50 | In this course, I am satisfied enough with this course to recommend it to others. | 1 | 2 | 3 | 4 | 5 |
| 51 | This course compared to face-to-face course settings; I am less satisfied with this learning experience. | 1 | 2 | 3 | 4 | 5 |
| 52 | In this course, I enjoy working on assignments by myself. | 1 | 2 | 3 | 4 | 5 |
| 53 | In this course, the instructor makes me feel that I am a true member of the class. | 1 | 2 | 3 | 4 | 5 |
| 54 | In this course, I am dissatisfied with the accessibility and availability of the instructor. | 1 | 2 | 3 | 4 | 5 |
| 55 | In this course, the instructor uses the flipped classroom approach appropriately. | 1 | 2 | 3 | 4 | 5 |
| 56 | In this course, class assignments were clearly communicated to me. | 1 | 2 | 3 | 4 | 5 |
| 57 | In this course, feedback on the evaluation of tests and other assignments was given in a timely manner. | 1 | 2 | 3 | 4 | 5 |
| 58 | In this course, the instructor’s voice is audible. | 1 | 2 | 3 | 4 | 5 |
| 59 | In this course, course content shown or displayed on the online platform is clear. | 1 | 2 | 3 | 4 | 5 |
| 60 | In this course, the video image is clear and comprehensive. | 1 | 2 | 3 | 4 | 5 |
| 61 | In this course, technical problems are not frequent, and they do not adversely affect my understanding of the course. | 1 | 2 | 3 | 4 | 5 |
| 62 | In this course, the technology used for the flipped classroom is reliable. | 1 | 2 | 3 | 4 | 5 |

**Part D: Online learning Anxiety**

| No | **In this course….** | SD | D | N | A | SA |
| --- | --- | --- | --- | --- | --- | --- |
| 63 | I feel apprehensive about using computers. | 1 | 2 | 3 | 4 | 5 |
| 64 | I fear of making mistakes I cannot correct. | 1 | 2 | 3 | 4 | 5 |
| 65 | I am worried if I can properly operate the system. | 1 | 2 | 3 | 4 | 5 |
| 66 | I am worried if I can understand computer-related terms. | 1 | 2 | 3 | 4 | 5 |
| 67 | I fear of hitting the wrong key on the keyboard or clicking the wrong hyperlink. | 1 | 2 | 3 | 4 | 5 |
| 68 | In this course, I am tense and nervous while participating in group discussions. | 1 | 2 | 3 | 4 | 5 |
| 69 | In this course, I am worried whether or not I can communicate effectively with other learners in online learning environment. | 1 | 2 | 3 | 4 | 5 |
| 70 | In this course, I fear of communicating with a new classmate. | 1 | 2 | 3 | 4 | 5 |
| 71 | In this course, I feel apprehensive about learning via the Internet. | 1 | 2 | 3 | 4 | 5 |
| 72 | In this course, I am worried whether or not I can accomplish the learning objective. | 1 | 2 | 3 | 4 | 5 |
| 73 | In this course, I am worried about my achievements in online cooperative learning. | 1 | 2 | 3 | 4 | 5 |
| 74 | In this course, I am worried whether or not I can gather the needed information. | 1 | 2 | 3 | 4 | 5 |
